# Prevalence of dermatoses in geriatric singaporeans in the community - a cross-sectional study

**DOI:** 10.1186/s12875-024-02525-y

**Published:** 2024-08-09

**Authors:** Lester Juay, Monil Nagad Bhupendrabhai, Siti Hafizah Binte Ahmad, Hung Chew Wong, Justin Wee-Min Chong, Wee Hian Tan, Nisha Suyien Chandran

**Affiliations:** 1 Division of Dermatology, National University Hospital, 5 Lower Kent Ridge Road, Singapore city, 119074 Singapore; 2 Department of Medicine, National University Hospital, Singapore city, Singapore; 3 Yong Loo Lin School of Medicine, National University of Singapore, Singapore city, Singapore; 4 National University Polyclinics, National University Health System, Singapore city, Singapore

**Keywords:** Asteatosis, Dermatology, Eczema, Elderly, Geriatric medicine, Primary care education, Singapore

## Abstract

**Background:**

Little is known about the prevalence of dermatoses in “skin-well” geriatric Singaporeans. We aim to identify the prevalence of dermatoses and their associations within the geriatric population in Singapore, and to understand the distribution of dermatological encounters presenting to primary care physicians, and the resultant referral behaviour.

**Methods:**

A joint quantitative-qualitative study was performed across 8 months. Patients aged 65 years and above who visited a local polyclinic for management of non-dermatological chronic diseases were recruited. They were administered questionnaires, and underwent full skin examinations. Online surveys were disseminated to polyclinic physicians under the same healthcare cluster.

**Results:**

201 patients and 53 physicians were recruited. The most common dermatoses identified in patients were benign tumours and cysts (97.5%), and asteatosis (81.6%). For every 1-year increase in age, the odds of having asteatosis increased by 13.5% (95% CI 3.4–24.7%, *p* = 0.008), and urticarial disorders by 14.6% (95% CI 0.3–30.9%, *p* = 0.045). Patients who used any form of topical preparations on a daily basis had higher odds of having eczema and inflammatory dermatoses (OR 2.51, 95% CI 1.38 to 4.56, *p* = 0.003). Physicians reported dermatological conditions involving 20% of all clinical encounters. Eczema represented the most commonly reported dermatosis within the first visit. 50% of dermatology referrals were done solely at the patient’s own request.

**Conclusion:**

The prevalence of dermatoses in the elderly in Singapore is high, especially asteatosis. Prompt recognition by the primary healthcare provider potentially prevents future morbidity. Outreach education for both primary care physicians and the general public will be key.

**Ethics approval:**

National Healthcare group (NHG) Domain Specific Review Board (DSRB), Singapore, under Trial Registration Number 2020/00239, dated 11 August 2020.

**Supplementary Information:**

The online version contains supplementary material available at 10.1186/s12875-024-02525-y.

## Background

Skin disease burden in the elderly is high. Aging leads to reduced functional capacity and increased susceptibility of the skin to dryness, itching, ulcers, dyspigmentation, wrinkles, infections, as well as tumours [1]. 

Descriptive studies on geriatric dermatoses so far are mostly done in countries where Caucasian demographic predominates. A study in the United Kingdom identified environmental factors (e.g. pollution), and lifestyle factors (e.g. smoking, sunbed use), which negatively affect skin health. The resultant dermatoses decrease general health and reduce the likelihood of healthy and graceful aging [1]. Mekic et al. demonstrated a 60% prevalence of dry skin among the 5547 middle-aged to elderly individuals of the Rotterdam Study (2018) [2]. 

Singapore has very different lifestyle habits and healthcare models, compared with many parts of the world. The Singaporean population is rapidly aging, with 40.1% aged 60 years and older by year 2050 [3]. A local retrospective descriptive study (1990) of 2,571 patients aged 65 years and older presenting as outpatients to the National Skin Centre indicated eczema accounting for 35.3% of all dermatoses, with asteatotic eczema being distinctly common [4]. The latest local data from the same centre is a 15-year cohort study (2022) which similarly indicated dermatitis as the most common dermatosis (37.7%) among the elderly [5]. However, these findings represented elderly who actively sought treatment for their skin conditions from a tertiary centre. A local multicentre cross-sectional study (2020) studying the prevalence of skin disease in residents of nursing care facilities showed (in order of decreasing prevalence) eczema, tinea infections, cellulitis, and scabies as the most common dermatoses within institutionalised residents [6]. Again, this study captured only the frail elderly population with generally poor functional status, and may over-represent the dermatoses found in majority of the healthier elderly.

A significant proportion of elderly patients in Singapore frequent their primary care physicians (PCPs) for routine management of their chronic medical conditions. These consults present as opportunities for PCPs to address any concomitant dermatological complaints which may be raised, or to opportunistically identify and treat any asymptomatic dermatoses which may subsequently deteriorate and increase morbidity. In England and Wales, skin conditions were the most common reason for a consult to primary care (24% of all new reasons for consult), while eczema was a common diagnosis in primary care dermatological consults (22.5%) [7, 8]. 

There remain insufficient data on the prevalence of skin conditions in the average geriatric population. This study aims to elucidate the local burden of skin conditions in the elderly, who are seemingly asymptomatic from a dermatological perspective (“skin-well”). We took special interest in those who frequented primary care facilities, as they represented the population for which dermatoses may remain undetected, despite multiple clinical encounters with their PCPs.

We aim to identify the prevalence of various dermatoses within the geriatric population in the local community who seek chronic medical care, and their possible associations with lifestyle habits, occupation, and other underlying medical conditions. Concurrently, we hope to understand the distribution of dermatological encounters presenting to PCPs, and their resultant referral behaviour.

## Methods

We performed a joint quantitative-qualitative study, where patients and PCPs were concurrently recruited from Pioneer Polyclinic (a public primary care clinic in Singapore) over a period of 8 months.

In the ‘patient arm’ of the study, patients aged 65 years and above who visited Pioneer Polyclinic for management of their chronic diseases were recruited. The study team would visit Pioneer Polyclinic on sessions that ran clinics predominantly for chronic consults. Those who intended to seek consult for skin conditions were excluded. Patients who consented for this study were administered a questionnaire (Annex 1), and then directed to a separate room for full skin examination by a dermatology senior resident (SR) or resident physician (RP) from National University Hospital (NUH), Singapore. Dermatoses diagnosed were subsumed within broad categories (Table 1). In the event of identification of any previously undiagnosed dermatoses, the dermatology SR or RP would inform the primary physician who first attended to the patient and suggest appropriate management plans. Had specialist services been required, a referral would be triggered to the NUH Division of Dermatology.

**Table 1:** Categories of dermatoses collected from the 201 patients encountered

Dermatosis	Examples of diagnoses within each category
Asteatosis	Asteatotic/xerotic eczema
Eczematous (excluding asteatotic eczema) and inflammatory dermatoses	Seborrhoeic dermatitis, intertrigo, atopic eczema, lichen simplex chronicus, stasis eczema/dermatitis, erythroderma, prurigo, callosity, lipodermatosclerosis, scalp dermatitis, psoriasis, acanthosis nigricans, contact dermatitis, lichen planus
Pigmentary disorders	Solar lentigo, ephelides, idiopathic guttate hypomelanosis, post inflammatory dyspigmentation, melasma, progressive macular hypomelanosis, cafe au lait macule, vitiligo
Nail diseases	Pincer nail deformity, onycholysis, subungual haematoma, onychomycosis, onychodystrophy/nail dystrophy, chronic paronychia, longitudinal melanonychia, ingrown toenail, subungual hyperkeratosis, pterygium
Vascular diseases	Varicose vein, spider vein, venous lake, capillary malformation, telangiectasia, lymphoedema, venous stasis
Naevi	Junctional naevus, intradermal naevus, compound naevus
Non-naevi benign tumours and cysts	Seborrhoeic keratosis, syringoma, skin tag, xanthelasma, cherry angioma, fibroepithelial polyp, haemangioma, lipoma, epidermal cyst, dermatofibroma, neurofibroma, angiokeratoma
Pre-malignant or malignant tumours	Basal cell carcinoma, actinic keratosis, cutaneous horn
Scars	Scar, hypertrophic scar, keloid
Hair disorders	Androgenetic alopecia, scarring alopecia
Pilosebaceous disorders	Seborrhoea, acne, comedones, sebaceous hyperplasia, milia, scalp folliculitis, keratosis pilaris
Purpura	Senile purpura, pigmented purpuric dermatosis
Depositional disorders	Cutaneous amyloidosis, macular amyloidosis, lichen amyloidosis
Cutaneous infections	Viral wart, tinea, pityriasis versicolor, ecthyma
Urticarial disorders	Urticaria
Ulcers	Venous ulcer, neuropathic ulcer, diabetic ulcer

The questionnaire provided information on the basic biodata of the patient, lifestyle habits, as well as simple medical history. Dermatoses identified during physical examination of the skin were documented. For conditions which could not be diagnosed within the clinical encounter without further investigations (e.g. suspected skin cancers), the relevant data would be retrospectively entered once the diagnoses were confirmed by the dermatologist. Biodata collected via the questionnaires were analysed against the prevalence of dermatoses diagnosed on physical examination.

In the ‘physician arm’ of the study, an online survey was concurrently disseminated to PCPs from polyclinics under the same healthcare cluster, for their voluntary participation. The survey was open over the same period of 8 months. The responses were anonymised, and no identifiers were collected.

The online survey for physicians (Annex 2) collected 2 biodata points, namely the age group, and the years of work experience. Special effort was made to avoid physicians disclosing their exact age, to minimise the chances of the respondent being easily identified. Clinical information based on the physicians’ recollection of patient encounters within the immediate past 2 weeks were collected and as follows:

- The approximate incidence of dermatoses as a primary reason of encounter.
- The approximate incidence of encountering various broad groups of dermatoses.
- The proportion of cases within each of the above broad groups of dermatoses which were managed and treated within the primary care setting, compared with the proportion of cases which were referred to the specialist within the first consult.

Descriptive statistics for numerical variables were presented as median, 25th percentile, 75th percentile, minimum and maximum values while number and percentage of observations were presented for categorical variables. Generalised linear models were carried out to analyse the association between factors of interest and the types of dermatoses, and also the rate of referrals between physicians of different years of work experience. The factors of interest were age, smoking status, personal and family history of cancers, consumption of beverages (specifically coffee, tea, and cola), sleeping in air-conditioned room, use of body creams or moisturisers on a daily basis, traditional or alternative medications and frequency of showers. Statistical analyses were performed using SAS version 9.4 (SAS Institute, Inc; Cary, NC, USA).

## Results

A total of 201 patients and 53 PCPs were recruited over the period of January to August 2021.

The patient-arm of the study revealed that patients were of age range of 65 to 92 years old. 68.2% of patients were male. 64.7% were Chinese in ethnicity, 22.4% were Malay and 12.4% were Indian. Notably, this is in keeping with the ethnic spread of Singapore [3]. The most common dermatosis picked up on physical examination was that of benign tumours and cysts, being found in 97.5% of patients. The second most common dermatosis was that of asteatosis, which was found in 81.6% of patients.

For every 1-year increase in age, the odds of having asteatosis increased significantly by 13.5% (95% CI 3.4–24.7%, *p* = 0.008), and the odds of having urticarial disorders increased significantly by 14.6% (95% CI 0.3–30.9%, *p* = 0.045). Patients who used any form of topical preparations, including moisturisers, on a daily basis had significantly higher odds of having eczema and inflammatory dermatoses (OR 2.51, 95% CI 1.38 to 4.56, *p* = 0.003).

There was otherwise no significant difference between patients with any specific dermatoses and smoking status, personal and family history of cancers, consumption of coffee, tea, or cola, sleeping in air-conditioned room, use of traditional or alternative medications, nor frequency of showers.

**Fig. 1:**
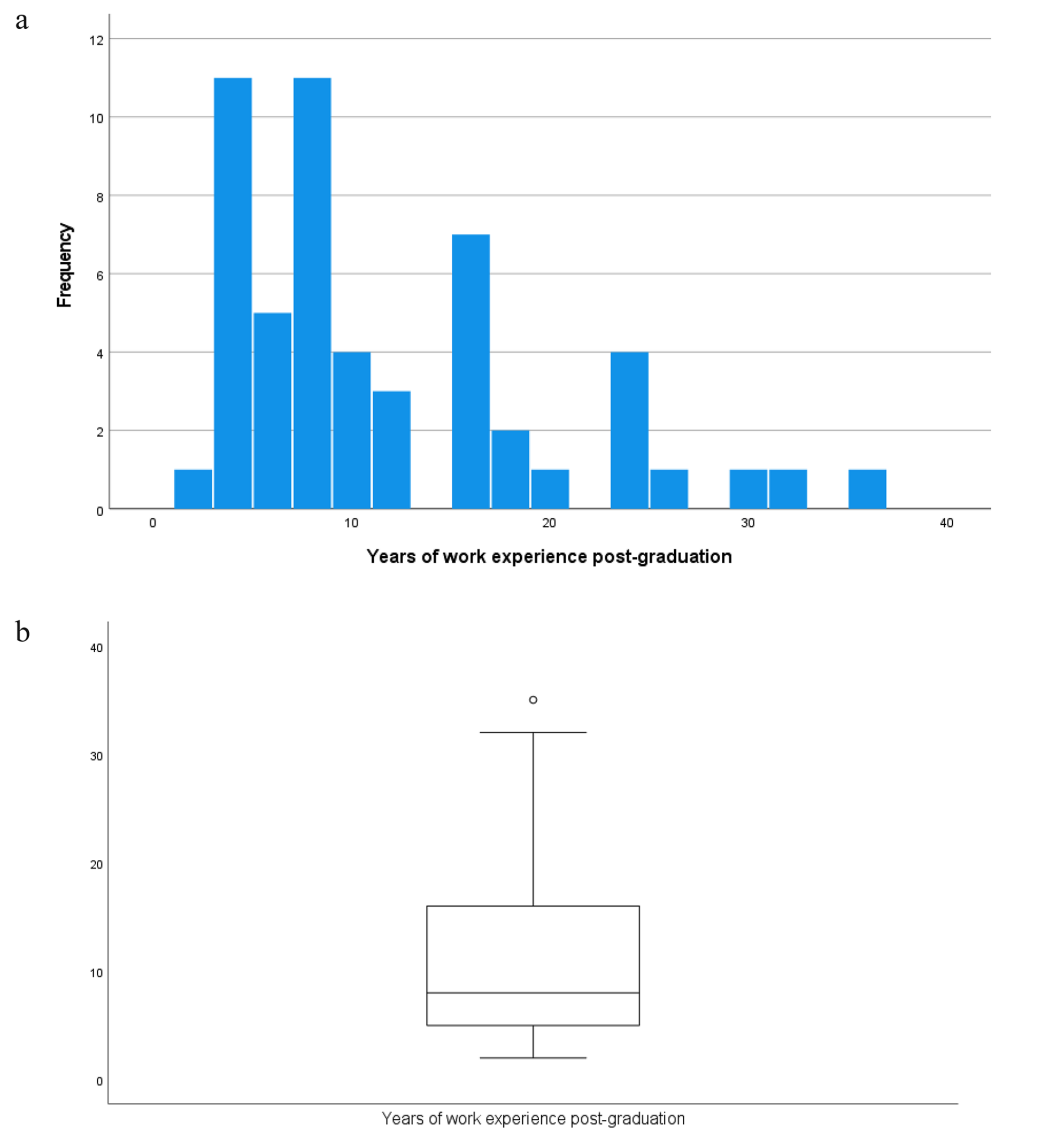
**a** Years of work experience post-graduation in the 53 physicians (represented in bar chart). **b** Years of work experience post-graduation in the 53 physicians (represented in box plot)

In the “physician arm” of the study, responses from 53 PCPs were analysed. The years of work experience post-graduation (Fig. 1a and b) indicate an almost equivalent distribution of those with less than 10 years of work experience and those with 10 years or more.

Physicians reported dermatological conditions involving 20% (median) of all clinical encounters, and 50% (median) of referrals to a dermatologist being done solely due to the patient’s own request (Table 2). Within a 2-week period, these 53 PCPs cumulatively reported approximately 333 cases of eczema as first visit encounters to the polyclinic, of which 64 cases (19.2%) were referred to specialist care within the first visit. The cumulative cases of other dermatoses, and the proportion referred to the specialist within the first visit, are shown in Table 3. There was no significant difference between the rate of eczema referrals between physicians of different years of work experience.

**Table 2:** Proportion of consults involving dermatological conditions, referrals on the first encounter, and referrals made solely due to the patient’s request

	*N* = 53
Proportion of consults (%), in the past week, consists of patients who have skin conditions as one of their complaints	
N Median (25th percentile – 75th percentile) Minimum – Maximum	53 20 (10–40) 2–75
Proportion of referrals (%) done solely due to the patient’s own request (that the PCP would otherwise have just managed in the primary care setting)	
N Median (25th percentile – 75th percentile) Minimum – Maximum	53 50 (10–65) 0–100

**Table 3:** Cumulative cases of dermatoses, and the proportion referred to the specialist within the first visit

Dermatoses	Cumulative Cases by 53 Physicians	Number and Percentage of Cumulative Cases Referred to Specialist within the First Visit
Eczema	333	64 (19.2%)
Psoriasis	36	17 (47.2%)
Exanthema	184	21 (11.4%)
Blistering dermatoses	43	8 (18.6%)
Pigmentary dermatoses	38	28 (73.7%)
Cutaneous infections	168	40 (23.8%)
Benign cutaneous lesions	182	76 (41.8%)
Malignant cutaneous lesions	11	11 (100%)

Eczema represented the most commonly reported dermatological condition encountered within the first visit. This was followed by 184 cases of exanthema, 182 cases of benign tumours and 168 cases of cutaneous infections. The 3 conditions most likely to be referred to dermatologists during the first primary care encounter were malignant cutaneous lesions (100%), pigmentary dermatoses (73.7%), and psoriasis (47.2%).

## Discussion

Our study protocol collected data from both patients and physicians to achieve multiple objectives concurrently. The results from the ‘patient arm’ of the study parallel known literature, demonstrating high rates of asteatosis affecting the geriatric population. Ageing leads to functional and structural alterations in the stratum corneum, which in turn causes skin to be more fragile, more prone to irritation, inflammation and infections [9].

Our study found that 81.6% of geriatric patients who presented for chronic medical care had asteatosis, and that the odds of having asteatosis increased by 13.5% with every additional year of age. Despite this high prevalence of asteatosis, these patients often do not seek treatment in early stages as they are largely asymptomatic. The cumulative healthcare burden of asteatosis can become significant, as asteatosis is a precursor for developing symptomatic asteatotic eczema. A review of our in-house data registry of outpatients seen by the Division of Dermatology, NUH, indicated a total of 4985 outpatient first visits in the year 2019, of which 1157 encounters (23.2%) were by those 65 years and older. 444 (38.4%) of these 1157 encounters were for eczema, which is much higher in contrast to cutaneous malignancies (9.7%). Furthermore, the same in-house data registry of 1277 inpatient encounters (in the form of interdepartmental referrals or dermatology inpatients) seen by the Division of Dermatology, NUH within the year 2019 indicated 390 (30.5%) patients seen for eczema alone, of which 201 (15.7% of total inpatient encounters, 51.5% of all inpatient eczema encounters) were geriatric patients with eczema. These inpatients tend to suffer from more severe forms of eczema (e.g. erythrodermic eczema), or complications of eczema, such as skin and soft tissue infections. This highlights the high burden of eczema of which asteatosis is a significant risk factor, especially in the geriatric population.

While our data suggests that the incidence of urticarial disorders increases with age, the in-house data registry indicates only 3.0% of first visit outpatient encounters and 104 (8.1%) of 1277 inpatient encounters being due to urticarial disorders. Only 33 (2.6%) inpatients were of the geriatric age group. Urticaria often presents in the acute form, which portends rapid resolution. This in turn reduces the number of patients who request for referrals to see specialists. In contrast, eczema is caused by a complex interplay of intrinsic and extrinsic factors which may not respond as readily to first-line therapy. Patients may be more inclined to perceive treatment failure and hence request for specialist review.

The positive correlation between the use of topical preparations and having eczema and inflammatory dermatoses likely represents an association more than a causation. As our questionnaire did not further sub-classify the nature of the topicals used, it raises a possibility of a bidirectional relationship between eczema and the use of topicals. We purport that the elderly with eczema were more likely to be symptomatic, and hence would have self-attempted topical therapy. However, we recognise that traditional salves or ointments containing salicylates, camphor, essential oils or other contactants, which are readily obtainable over the counter in Singapore, may cause contact dermatitis, accounting for eczema in these patients as well. The cross-sectional nature of this study limits elucidation of this aspect. Future local studies can aim to better characterise the exact nature of topical usage in the average Singaporean elderly.

The methodology of examining “skin-well” elderly is a strength, compared to most existing Singaporean studies which drew data retrospectively from tertiary care institutions and national registries [4, 6]. Our patient population more accurately represents the average skin-well elderly Singaporean population at large, and as a result, reduces surveillance bias and an overrepresentation of dermatoses. For example, no cases of scabies were detected in our skin-well population. Scabetic infestations would tend to be symptomatic, and affected patients would likely seek medical help. Conversely, our current methodology allows us to identify asymptomatic dermatoses which may exert clinically important downstream effects if left untreated.

The finding of approximately 20% of clinical encounters reported by our surveyed PCPs as being related to dermatological disorders is consistent with published prevalence from England and Wales [7], suggesting that the high dermatological disease burden transcends communities of all skin types and locations. However, Singapore possesses unique geographic factors and healthcare models which in turn shape and alter the behaviour of the average patient. As a small and densely populated city-state, Singapore has tertiary hospitals within close proximity to all residential areas of the country; most residents have access to these hospitals within a 30-minute commute. This greatly lowers the physical distance barrier to specialist visits, which may be an important deterrent in other larger nations. Furthermore, the Singaporean healthcare model historically has strong specialist presence in the holistic care of an individual. This translates to patients having a low threshold to seek specialist review, and may explain the finding of how our surveyed physicians reported 50% of their first visit referrals being done solely due to the patient’s own request.

Malignant and benign cutaneous lesions, psoriasis and pigmentary disorders were reported by PCPs as being referred at a higher rate on the first visit. A qualitative study in the United Kingdom indicated that current routine practice for psoriasis management in primary care was mismatched with the expressed needs of patients [10]. Patients perceived PCPs to lack confidence in the assessment and management of psoriasis, and both patients and physicians felt lacking in knowledge about the condition. A Spanish study (2012) looking at the reasons for primary care referral to dermatology and diagnostic agreement between PCPs and dermatologists analysed data from 755 patients with 882 first visit encounters in a span of 3 months; the most common diagnoses included benign cutaneous conditions such as seborrhoeic keratoses and melanocytic naevi [11]. Primary care diagnoses for skin tumours was reported to have a sensitivity of 22.4%, and a specificity of 94.7%; this meant that PCPs were better qualified to rule out a given skin condition (high specificity) than to establish an accurate clinical diagnosis (poor sensitivity). This may mirror the situation in Singapore as well, where PCPs more readily refer cutaneous lesions to rule out malignancy.

A limitation of the ‘patient arm’ of the study was the small sample size. Oh et al. published a large scale prospective study of 63,257 men and women who were 45 to 74 years old at recruitment from 1993 to 1998 [12], which demonstrated that caffeine intake reduced non-melanoma skin cancer risk in a dose-dependent manner. We did not find a significant relationship between skin cancers and caffeine intake, as well as known risk factors of sun exposure or smoking, in our own data set. This is mostly likely driven by an underpowered study to pick up rarer diagnoses.

We recognise potential selection bias in our patient recruitment process, where patients with self-perceived skin issues may have been more forthcoming with volunteering for recruitment and examination. In addition, patient recruitment occurred during the COVID pandemic. This might have resulted in some ‘skin-well’ elderly intentionally declining skin examination to minimise unnecessary human contact during their visit to a healthcare facility. To minimise this bias, we propose future studies involving PCPs performing full skin examinations as part of their chronic health checks for asymptomatic elderly patients.

In the United Kingdom, there has been recognition of insufficient dermatological exposure in medical school, where students receive no more than 6 days training in dermatology, but yet a quarter of primary care appointments concern the skin [13]. A recent United Kingdom cross-sectional study of 318 video-recorded consultations identified 14.2% of consults pertaining to 1 or more dermatological problems, of which there was low shared decision making and infrequent self-management advice given to the patients. A striking finding of the study highlighted that while 84.3% of skin problems were not referred to secondary care, 32.6% of those not referred were seen again in primary care within 12 weeks, of which 35.7% follow-up appointments were unplanned [14]. This study demonstrated the challenges of dermatological care in the primary care setting, and the authors postulated that the complexities of juggling multiple presenting complaints within time-limited consultations led to less than ideal counselling and follow-up accorded to the dermatological conditions. A qualitative study (2007) carried out in the United Kingdom compared the effectiveness of a “General Practitioner with Special Interests” (GPSI) dermatology service with standard consultant-led dermatology outpatient care [15]. The study surmised that GPSI services were acceptable to the majority, but there was likely to be a group of patients with longstanding, though clinically non-urgent, conditions for whom the service would not be acceptable. From referral pattern data of our study, psoriasis and pigmentary disorders can perhaps be prioritised by dermatologists for outreach education to PCPs on effective first-line management and referral thresholds, to improve right-siting of care. Specialised workflows that streamline diagnosis and management of cutaneous lesions can improve patient experience and reduce burden on the limited resources in public hospitals. Healthcare clusters in Singapore likewise can train and employ GPSIs in dermatology, with aims to institute better preventive and early dermatological care.

## Conclusion

The prevalence of skin conditions in the elderly visiting public primary healthcare institutions was high, especially asteatosis. Prompt recognition by PCPs and early treatment can potentially prevent future burden as a result of progression of skin disease.

### Electronic supplementary material

Below is the link to the electronic supplementary material.

Supplementary Material 1

Supplementary Material 2

## Data Availability

Datasets can be requested from the corresponding author, Lester Juay, at lester_juay@nuhs.edu.sg.
