# Supplementary material for: Prevalence of dermatoses in geriatric singaporeans in the community - a cross-sectional study
Source: BMC Prim Care. 2024 Aug 9;25:290. doi: 10.1186/s12875-024-02525-y (PMC11312226; doi:10.1186/s12875-024-02525-y)
Supplement: Supplementary file 1 — Supplementary Material 1 [file 12875_2024_2525_MOESM1_ESM.docx]

**Questionnaire for Study on “Prevalence of Dermatoses in Geriatric Singaporeans in the Community - A Cross-Sectional Study” for Patients**

**Age: __**

**Sex:** F / M

**Race:** Chinese / Malay / Indian / Others

1. **What is your occupation / What was/were your previous occupation(s)? ______**
   1. **Approximately what proportion of the work was outdoors?**

( Hardly outdoors / somewhat equivalent amount of indoors and outdoors / often outdoors )

1. **Do you smoke?**

(Never a smoker / Still smoking / Ex-smoker)

- 1. **At what age did you start smoking?** ___
  2. **If you have stopped smoking, at what age did you stop smoking?** __
  3. On average, since the start of smoking, **how many sticks a day did you smoke?** __

1. **Have you ever been diagnosed with cancer?** ( Yes / No )
   1. If yes, **are you actively undergoing any form of cancer treatment**? ( Yes / No )
   2. If yes, please state the **type(s) of cancer you have been diagnosed with**: ___
2. **Has anyone in your family been diagnosed with cancer?** ( Yes / No )
   1. If yes, **please state the relationship with the relative involved**: __
   2. If yes, please state the **type(s) of cancer they have been diagnosed with**: ___
3. **What is your preferred mobility aid at home?**

( I do not use any aids / Walking stick / Walking frame / Motorised scooter / Wheelchair / Bedbound )

1. **What is your preferred mobility aid outside of home?**

( I do not use any aids / Walking stick / Walking frame / Motorised scooter / Wheelchair / I do not leave home )

- 1. **How much time** (in hours) **do you spend outside of home?** ___

1. **How often do you drink the following beverages?**
   1. **Coffee** ( I do not drink coffee / less often than 1 cup a day / 1 cup a day / 2 cups a day / more often than 2 cups a day )
   2. **Tea** ( I do not drink tea / less often than 1 cup a day / 1 cup a day / 2 cups a day / more often than 2 cups a day )
   3. **Cola, or any carbonated canned drinks** ( I do not drink carbonated canned drinks / less often than 1 can a day / 1 can a day / 2 cans a day / more often than 2 cans a day )
2. **How often do you shower a day?** ( Less than once a day / once a day / twice a day / more than twice a day)
   1. Approximately **how long** (in minutes) **do your showers last for?** ____
   2. **Do you shower with heated water**? ( Yes / No, I shower with room temperature water )
3. **Do you sleep in an air-conditioned room at night?** ( Yes / No )
4. **Do you use body creams / moisturisers on a daily basis?** ( Yes / No )
   1. If yes, **are these creams / moisturisers prescribed by the doctor?** ( Yes / No )
5. **How often do you travel overseas via air, within the span of one year? __**
   1. If yes, **list the travel destinations (by country, and time of year),** within the past one year ___
6. **Do you have a caregiver at home?** ( Yes / No )
   1. If yes, **which of the following is your caregiver?** ( Family member / helper for the entire household / dedicated helper just for you )
7. **Do you use traditional/alternative medications?** ( Yes / No )
   1. If yes, **do you use traditional/alternative topical medications** (e.g. creams, balms, medicated baths, powder)**?** ( Yes / No )
   2. If yes, **do you use traditional/alternative oral medications** (e.g. pills, brewed herbs / powders)**?** ( Yes / No )
8. Approximately **how often do you visit the following, within the span of one year?**
   1. **GPs / polyclinic doctors**: __
   2. **Specialists in hospitals / private clinics**: __
   3. **Traditional Physicians** (e.g. Chinese, Ayurvedic): __
   4. **Spa / Beauty Parlours**: __
   5. **Massage Parlours**: __
