# Supplementary material for: Prevalence of dermatoses in geriatric singaporeans in the community - a cross-sectional study
Source: BMC Prim Care. 2024 Aug 9;25:290. doi: 10.1186/s12875-024-02525-y (PMC11312226; doi:10.1186/s12875-024-02525-y)
Supplement: Supplementary file 2 — Supplementary Material 2 [file 12875_2024_2525_MOESM2_ESM.docx]

**Questionnaire for Study on “Prevalence of Dermatoses in Geriatric Singaporeans in the Community - A Cross-Sectional Study” for Physicians**

**Age Range: 21-29, 30-39, 40-49, 50-59, 60 and above**

**Years of work experience post-graduation (rounded off to nearest year): ___**

*Please assist with answering the following questions, according to your own subjective encounters as a NUP Primary Care Physician.*

1. **What proportion of consults, in the past week, consists of patients who have skin conditions as one of their complaints?** Please give your answer as a percentage (between 0 to 100%). **__**
2. **Of the above patients for whom a specialist referral was made, what is the approximate proportion for whom this was done solely due to the patient's own request (that you would otherwise have just managed in the primary care setting)?** Please give your answer as a percentage (between 0 to 100%). **__**
3. **Please fill in the empty spaces in the table below, based on the approximate number of cases you had encountered within the past TWO WEEKS:**

| ***Category of skin conditions*** | Number of cases seen as a ***first clinical encounter***^1^? | Number of cases seen as a ***recurrent clinical encounter***^2^? | Number of cases referred to specialist on the ***first clinical encounter***^1^ for the skin condition? |
| --- | --- | --- | --- |
| **Eczema**  (e.g. atopic eczema, asteatotic eczema, contact dermatitis) |  |  |  |
| **Psoriasis**  (e.g. chronic plaque psoriasis, nail and scalp psoriasis) |  |  |  |
| **Exanthema**  (e.g. maculopapular eruptions) |  |  |  |
| **Blistering skin conditions**  (e.g. bullous pemphigoid) |  |  |  |
| **Pigmentary skin conditions**  (e.g. vitiligo, melasma) |  |  |  |
| **Cutaneous infections**  (e.g. viral wart, zoster, scabies) |  |  |  |
| **Benign cutaneous lumps**  (e.g. seborrhoeic keratosis, epidermal cyst, skin tags) |  |  |  |
| **Malignant skin conditions**  (e.g. SCC, melanoma) |  |  |  |

***** ”**First clinical encounter**” is defined as the first presentation to the physician for the condition. This includes either first visits to the acute clinics with the skin condition as a complaint, or the first time the skin condition is raised up, or identified by the physician, in the review clinics.

** “**Recurrent clinical encounter**” is defined as any follow up encounter which follows a previous encounter for the same skin condition.
